# Mono-Exponential Fitting in T2-Relaxometry: Relevance of *Offset* and First Echo

**DOI:** 10.1371/journal.pone.0145255

**Published:** 2015-12-17

**Authors:** David Milford, Nicolas Rosbach, Martin Bendszus, Sabine Heiland

**Affiliations:** Department of Neuroradiology, University Hospital Heidelberg, Heidelberg, Germany; University of Chicago, UNITED STATES

## Abstract

**Introduction:**

T2 relaxometry has become an important tool in quantitative MRI. Little focus has been put on the effect of the refocusing flip angle upon the *offset* parameter, which was introduced to account for a signal floor due to noise or to long T2 components. The aim of this study was to show that B1 imperfections contribute significantly to the *offset*. We further introduce a simple method to reduce the systematic error in T2 by discarding the first echo and using the *offset* fitting approach.

**Materials and Methods:**

Signal curves of T2 relaxometry were simulated based on extended phase graph theory and evaluated for 4 different methods (inclusion and exclusion of the first echo, while fitting with and without the *offset*). We further performed T2 relaxometry in a phantom at 9.4T magnetic resonance imaging scanner and used the same methods for post-processing as in the extended phase graph simulated data. Single spin echo sequences were used to determine the correct T2 time.

**Results:**

The simulation data showed that the systematic error in T2 and the *offset* depends on the refocusing pulse, the echo spacing and the echo train length. The systematic error could be reduced by discarding the first echo. Further reduction of the systematic T2 error was reached by using the *offset* as fitting parameter. The phantom experiments confirmed these findings.

**Conclusion:**

The fitted offset parameter in T2 relaxometry is influenced by imperfect refocusing pulses. Using the *offset* as a fitting parameter and discarding the first echo is a fast and easy method to minimize the error in T2, particularly for low to intermediate echo train length.

## Introduction

T2 relaxometry is a frequently used method of magnetic resonance imaging (MRI), particularly in preclinical and clinical research. Ever since the first publication in 1971 by Damadian [1], researchers and clinicians alike tried to determine the T2 as bio markers for various diseases and as a parameter for prognosis and therapy control.

The “gold standard” method for acquiring T2 relaxometry data is the use of multiple single Spin Echo (SE) sequences with different echo times (TE)[2]. Due to the time constraints in clinical routine, however, Multi-Spin Echo (MSE) sequences [3] are generally used. MSE allows for multiple echoes within one acquisition depending on the number of 180° refocusing pulses. The number of echoes is given by the so called echo train length (ETL) and is usually constructed as a CPMG sequence [4]. Major reasons for incorrect T2 times are imperfect slice excitation profiles and issues with B1 inhomogeneities yielding low refocusing flip angles (FA) [5–7]. Multiple groups have tried to compensate and correct for these inhomogeneities. These techniques, however, are usually computationally intensive, complicated to implement or are restricted to a certain set of sequence parameters [8–12]. Further reasons for inaccurate T2 measurements are long superimposing T2 components either due to partial volume effects or due to several proton pools [13, 14]; furthermore incorrect sampling of the signal decay can contribute to errors in T2 [15–17].

Although those potential sources of systematic errors in T2 calculation are known, more often than not, post-processing and data-fitting techniques do not account for them. Data from T2 relaxometry are most often fitted to a simple exponential curve:

$$S(TE)=kS_{o}.\exp(-TE/T2)$$ (1)

Where k is a proportionality constant subsuming signal gain or attenuation by the scanner’s hard-/software, S_o_ is the proton density and TE is the echo time. In most cases k and S_o_ are merged together to a single factor, because true proton density is hard to separate from the signal gain caused by the measurement process itself.

Besides this simple mono-exponential fit, sometimes an offset or baseline is introduced:

$$S(TE)=kS_{o}.\exp\left(-\frac{TE}{T2}\right)+offset,$$ (2)

where *offset* is thought to represent a non-zero baseline taking into account signal that may not have converged towards zero. While this approach is sometimes used without explanation for the non-zero baseline [18], some groups use it for compensation of long T2 components such as CSF [14, 19], and some use it to compensate for offset signal originating from the system [20] or Rican noise, particularly at low signal-to-noise (SNR) level [21, 22].

The aim of this study is to show, that B1 inhomogeneities and imperfect refocusing pulses contribute significantly to the *offset*. By using extended phase graph (EPG) theory [23–26] we aim to show that errors in the early echoes caused by B1 inhomogeneities increase the offset depending on FA, echo spacing (ESP) and echo train length (ETL) and the T2 of the tissue. We further introduce a simple method to reduce the systematic error on T2 due to B1 imperfection by discarding the very first echo and using the offset within the fitting approach.

## Materials and Methods

### Data simulation

T2 relaxation curves were simulated based on the EPG method [26]. Before one can answer which equation provides with the most stable solution, one has to know what parameters will change the signal and cause discrepancies in the evaluation of T2, S_o_ and offset. When simulating the data with EPG theory we have the ability to alter the following parameters; T2, T1, ESP, ETL, FA and S_o_. It has been shown in previous work that T1 does not alter the curves to a discernible degree [11]. Thus, we kept T1 constant at 3000ms. Unless otherwise stated the following parameters were used: S_o_ = 1000a.u., T2 = 100ms, ETL = 20–50ms in steps of 2ms, ESP = 10–40 in steps of 2 and FA = 120°-180° in steps of 20°. To monitor the accuracy of the four fitting methods for different T2, simulation were undertaken with T2 times of 20ms, 60ms, and 100ms. For these simulations we used an ESP time of 5ms with an ETL of 32 (to ensure that the complete T2 decay is covered by the echoes) and a FA of 120°. Furthermore, to monitor the effect of noise, the simulations were rerun with added Rican noise. The used algorithm to include the noise was S_r_ = F*Ra+S_c_, where S_r_ is the resulting signal with noise, F the noise factor (signal to noise ratio of the first echo, set to 10), Ra random generated number and S_c_ is the noise-free signal from the EPG simulation. These simulation were ran 1000 times to monitor not only the systematic error but the statistical error as well.

It is important to note that the EPG algorithm itself does not account for B1 inhomogeneity within the slice profile. One could follow the approach of Lebel et. al. [12] and break up the slice into partitions with quasi-homogeneous B1 and then perform EPG simulation for each partition. However, as the scope of this study is to identify the link between B1 inhomogeneity and *offset*, there is no need to distinguish between different sources of B1 inhomogenieties. Therefore EPG was used to simulate the measured signal for imperfect refocusing pulses without considering the origin of this imperfection.

### Phantom measurements

To test the results from the EPG based simulations, phantom measurements were performed using a 50ml tube filled with a 2.5% agarose to water mixture(to ensure that 3*T1 < TR). Images were performed on a Bruker 9.4T horizontal bore NMR scanner (BioSpec 94/20 USR, Bruker BioSpin GmbH, Ettlingen, Germany) with a four channel phased array surface coil. Firstly, spin echo sequences with single refocusing pulse were acquired to obtain the T2 time without systematic errors by B1 inhomogeneities. Following this, MSE sequences were used at different pulse angles (120°, 140°, 160° and 180°). The following parameters were the same in all spin echo and MSE sequences: TR = 3000ms, matrix = 150 x 150, FoV = 30mm x 30mm, slice thickness/number = 2mm/1, slice selective pulses, acquisition time = 7min 30sec). We performed 45 different spin echo sequences with TE ranging from 10ms to 450ms with 10ms spacing. For the MSE, we used ESP = 10ms and ETL = 45 to obtain the same echo times as used for the spin echo sequences.

### Calculation of T2

Curve fitting was undertaken in MATLAB, release 2014a® (MathWorks Inc.), using the Levenberg-Marquardt nonlinear least squares algorithm[27] provided by levmar [28]. Four different techniques where used for determining T2, S_o_ and the offset:

1. all echoes were fitted with Eq 1,
2. all echoes were fitted with Eq 2,
3. the first echo was discarded and the remaining echoes were fitted with Eq 1,
4. the first echo was discarded and the remaining echoes were fitted with Eq 2.

To determine the systematic error coming from curve fitting the relative deviation was calculated for T2 (dT2 = |(T2_fit_-T2_in_)|/T2_in_)*100) and S_o_ (dS_o_ = |(S_ofit_-S_oin_)|/S_oin_)*100).

## Results

### Influence of B1 Inhomogeneities upon *offset*

The offset parameter in Eq 2 has been introduced to cover cases where the T2 decay does not tend towards zero, but to an asymptote > 0. Due to the fitting process, however, *offset* is also influenced by errors caused by B1 inhomogeneities. More specifically *offset* equals the mean vertical offset from the measured data point to the fitted function:

$${offset}_{calc}=\frac{\sum_{i=1}^{i=ETL}\left(m_{i}-r_{i}\right)}{N}$$ (3)

where *r*_*i*_ is the signal from the fitted curve (using Eq 1) at the echo i, *m*_*i*_ is the measured or simulated signal, and N is the number of echoes.

**Fig 1:**
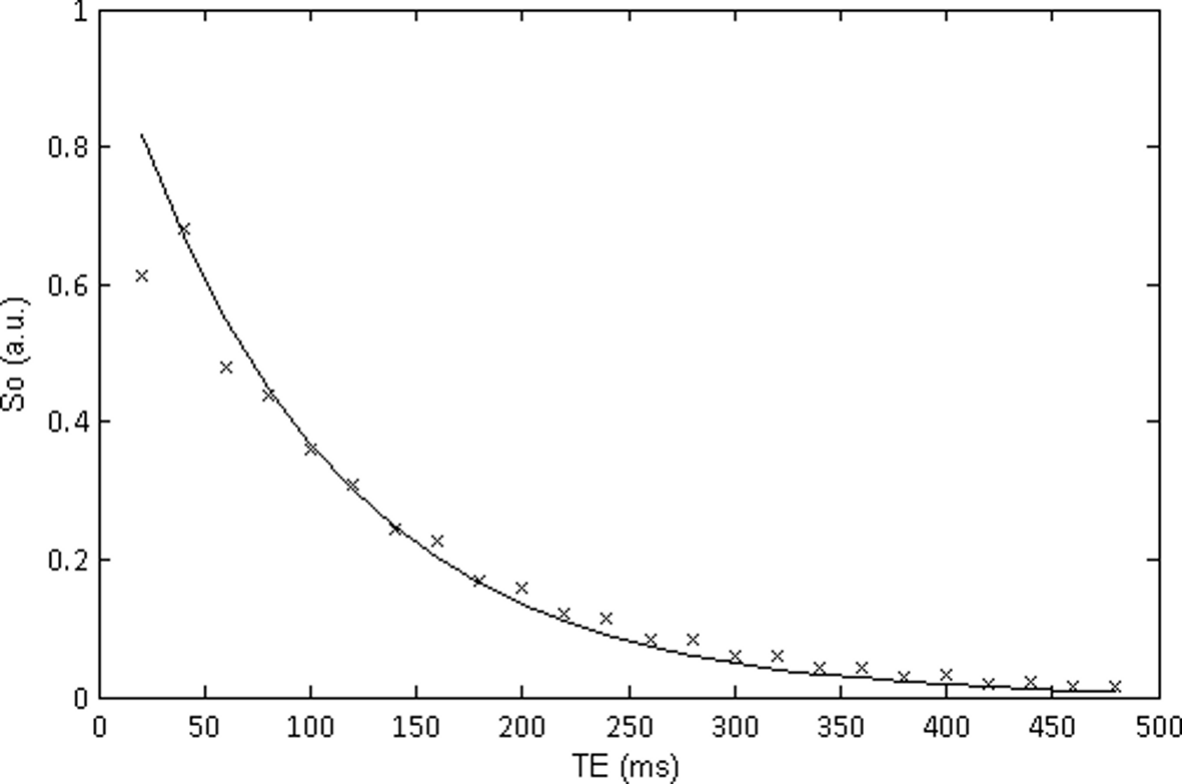
Illustration of a EPG derived curve with an FA of 120° (x) and correct 180° FA (line) for a T2 = 100ms, T1 = 3000ms, ESP = 20ms and ETL of 24. It can be seen that due to the incorrect FA the first echo point is lower than that of the second and the signal seems to oscillate between odd and even echoes.

To illustrate this, we generated a MSE signal curve based on EPG using the following parameters: T2 = 100ms, ESP = 20ms, ETL = 24 (Fig 1). FA is varied from 100° to 180° in steps of 10. The curve is then fitted as described above using Eq 2 to produce offset_fitted_. In a next step, offset_calc_ is calculated according to Eq 3 using the previously determined T2 and S_o_.

**Fig 2:**
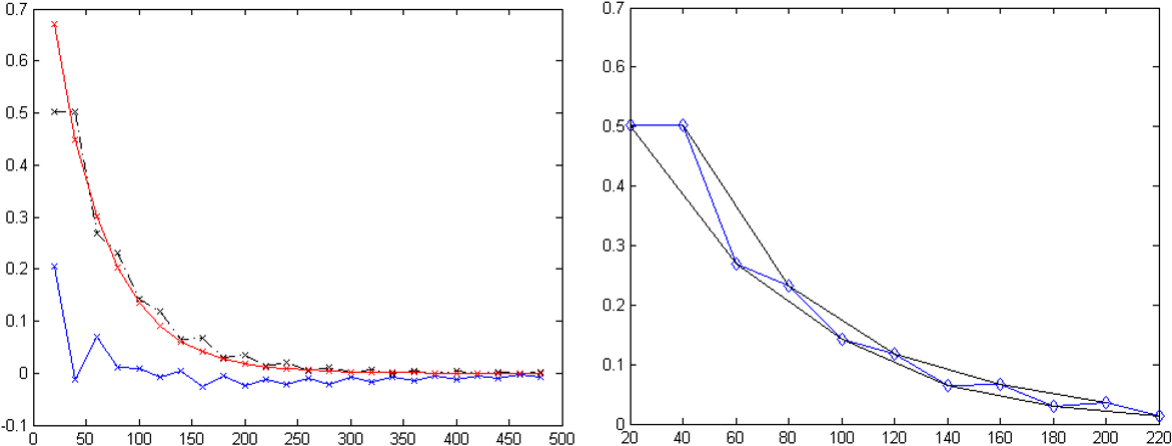
EPG simulated curve for T2 = 50ms, T1 = 3000ms, ESP = 20, ETL = 24, and FA = 120°. Fig **2**A illustrates the difference (blue solid line) between the curve simulated at 120° (black dashed line) an optimal 180° pulse (red solid line). Fig **2**B illustrates the envelope (black lines) calculated from the odd and even echoes of the signal (blue line).

Table 1 shows the results of these calculations. It can be seen, that although the simulated data converges to 0 for all FAs (Fig 1), the offset increases with decreasing FA. Furthermore offset_fitted_ equals offset_calc_ for all FA. This shows that the mean of the vertical offsets from the determined signal to the actual signal over all echoes substantially influences the offset. The main contribution to the offset, when it is of a higher magnitude, comes from the errors in the early echoes if the pulse angle is not a perfect 180°. This can be seen from Fig 2, where the first echo shows by far the largest difference from the ideal curve (at 180°) if measured at 120°. The error, which is also seen as an oscillation between odd and even echoes, decreases with increasing TE. It should also be noted that if noise was included in this signal the offset itself would not result as zero even for perfect refocusing due to the noise floor.

**Table 1:** Fitted results (T2_fitted_, So_fitted_, *Offset*_fitted_) from EPG simulated data of different FAs (T2 = 100ms, ESP = 20ms, ETL = 24, S_o_ = 1000 a.u.). ***Offset***_**calc**_ is the back calculated vertical offset (Eq 3).

FA (°)	T2_fitted_ (ms)	So_fitted_ (a.u)	offset_fitted_ (a.u)	offset_calc_ (a.u)
100	156.45	689.03	-20.61	-20.61
110	139.67	753.11	-14.86	-14.86
120	127.23	812.74	-10.41	-10.41
130	117.90	866.34	-6.92	-6.92
140	110.97	912.47	-4.25	-4.25
150	105.95	949.86	-2.26	-2.26
160	102.56	977.42	-0.89	-0.89
170	100.61	994.31	-0.16	-0.16
180	100.00	1000.00	0.00	0.00

### Comparison of different fitting methods: Simulation based on EPG

**Fig 3:**
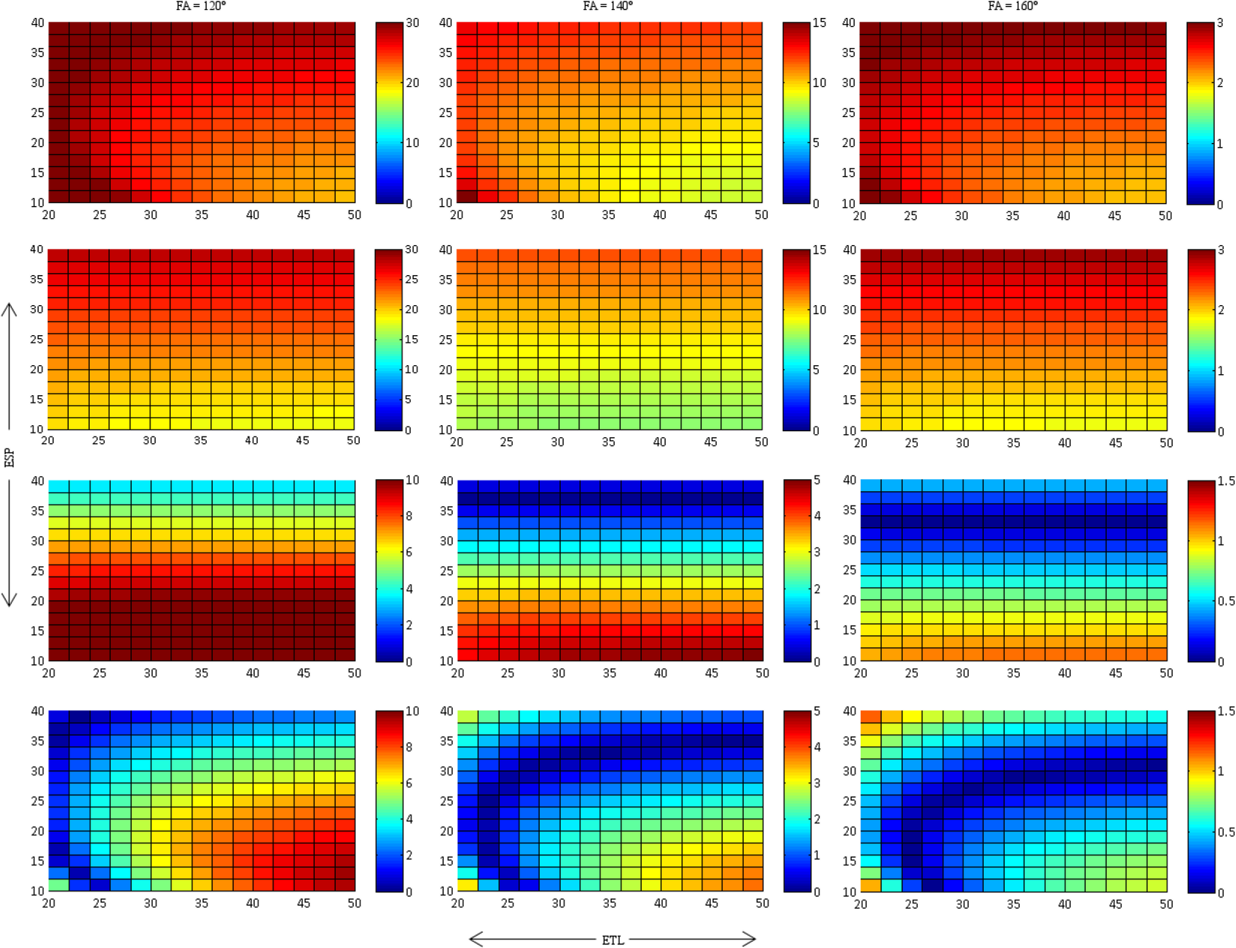
Relative T2 deviation, dT2 (in %), for method 1 (first row), method 2 (second row), method 3 (third row) and method 4 (forth row). These results are presented for 3 different FA. It is seen that T2 becomes longer as the FA decreases. Closer approximation to the actual T2 are seen when Eq 2 is used and the first point is excluded. Please note that the scales of dT2 are not uniform provide maximum dynamic range for the different ETL and ESP.

**Fig 4:**
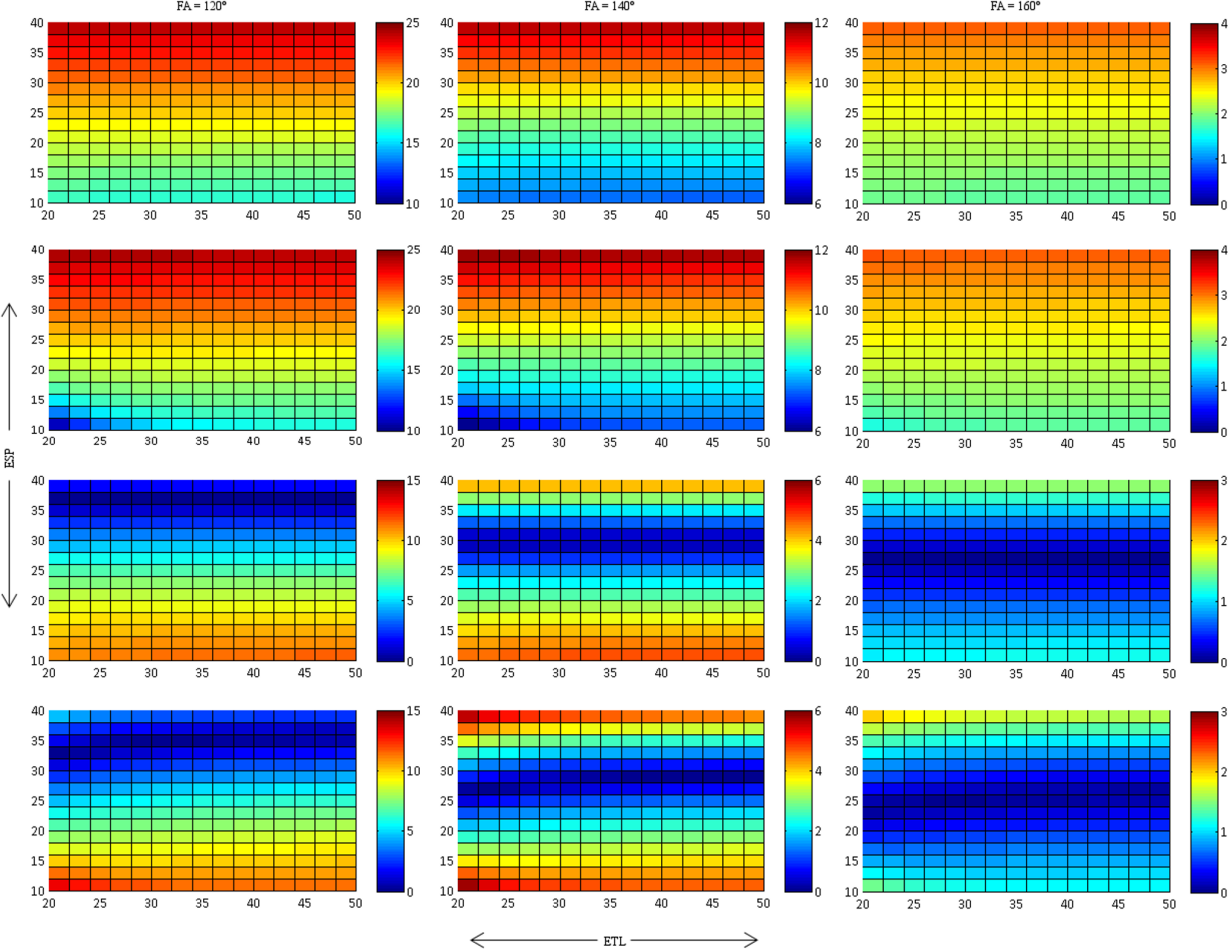
Relative S_0_ deviation, dS_0_ (in %), for methods 1–4 corresponding to rows 1–4 respectively. Closer approximations with little difference are seen for both equations with the first point excluded from the fit. Please note that the scales of dS_0_ are not uniform and therefore provide maximum dynamic range for the different ETL and ESP.

**Fig 5:**
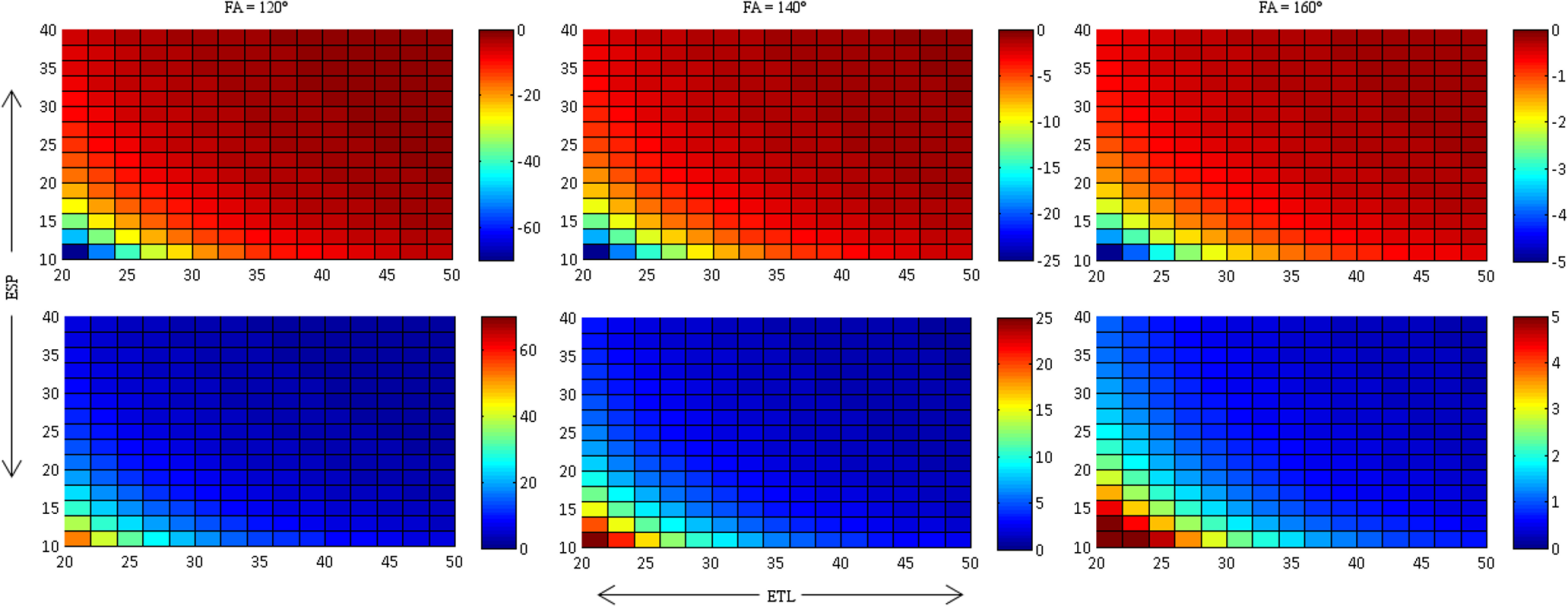
Determined offset values for different FAs for method 2 (top row) and method 4 (bottom row). Distributions remain relatively similar with decreasing values as FA tends to 180°. Note the decreasing offset with increasing FA.

For EPG simulations using 180° as refocusing angle, T2 and S_0_ determined after fitting matched the inputs for T2 and S_o_ exactly for all fitting methods. With method (2) and (4), where the offset is a fitting parameter, offset was 0 for all ETL an ESP. Thus, the plots for FA = 180° have not been included in Figs 3–5.

Fig 3 presents the relative deviation δT2 of the fitted T2 for the different fitting methods. There is a distinct increase ofδT2 with decreasing FA; δT2 at 120° is roughly a factor of 10 higher than δT2 at 160°. Furthermore, δT2 decreases for all FA, if the first echo is discarded (fitting methods (3) and (4)). The degree of reduction in δT2, however, depends on ETL and ESP: The lowest reduction in δT2 is seen for low ESP (method 3) and for low ESP and high ETL (method 4). With method 1, δT2 is highest for high ESP, whereas it is lowest for high ESP with method 3. In contrast, δT2 is lowest for high ETL and low ESP when using method 2, whereas it is highest at for high ETL and low ESP with method 4. While the introduction of the offset as fitting parameter does even slightly increase δT2 when using all echoes (method 2 vs. method 1), it leads to a gross decrease of δT2 over the majority of ESP/ETL combinations (method 4 vs. method 3). This decrease is most pronounced for low to intermediate ETL.

Fig 4 presents the relative deviation δS_0_ of the fitted S_0_ for the different fitting methods. As with δT2, δS_0_ increases markedly with decreasing FA. Discarding the first echo leads to a more accurate determination of S_0_ (i.e. δS_0_ decreases) for high to intermediate ESP, whereas there is little or no reduction of δS_0_ for low ESP. Other than with δT2, neither ETL nor the use of the offset as fitting parameter have a distinct influence on the accuracy of S_0_.

Fig 5 presents the determined offsets for the fittings with and without the first echo. Again, it is clear to see that the offset is heavily influenced by the FA: The more the FA deviates from 180°, the higher the offset. The highest offset is seen for low ETL and low ESP. Discarding the first echo has little effect on the absolute value of the offset other than sign reversal: Offset is negative, if the first echo is used (Fig 5, upper row), whereas it is positive, if the first echo is discarded (Fig 5., lower row). This shows, that the large error coming from the relative signal increase of the second echo with respect to the first echo (cf. Fig 2A), which is highest for combination of low ETL and ESP, has a huge influence on the offset and leads to an overcompensation, if method (2) is used for fitting and the first echo is not discarded. If the first echo is discarded, however, the offset helps to compensate for the oscillation between odd and even echoes. Therefore, using the offset as fitting parameter and discarding the first echo is particularly helpful for accurate determination of T2 if short echo trains are used (i.e. low ETL and low ESP).

### Comparison of different fitting methods: Influence of different T2 times and noise

Method four was shown to have the closest approximation to the actual T2 time for T2 = 100ms. In order to evaluate whether this result is still holds for different T2s the simulation where rerun. Table 2 presents the results for each method for the different T2. The results show again that method four, i.e. discarding the first echo and including the offset as fitting parameter, yield a closer approximation in comparison to the other techniques.

**Table 2:** Mean results of T2 fitting for each method per T2 time for an ESP time of 5ms (ETL = 32, FA = 140°). Simulations were ran 1000 times.

**Method 1**			**Method 2**		
**T2(ms)**	**T2**_**fitted**_ **(ms)**	**dT2 (%)**	**T2**_**fitted**_ **(ms)**	**dT2 (%)**	**Offset**
**20**	21.98	9.90	22.16	10.79	-1.67
**60**	64.54	7.57	66.22	10.37	-8.95
**100**	107.29	7.29	112.92	12.92	-25.04
**Method 3**			**Method 4**		
**T2(ms)**	**T2**_**fitted**_ **(ms)**	**dT2 (%)**	**T2**_**fitteds**_ **(ms)**	**dT2 (%)**	**Offset**
**20**	20.52	2.60	20.28	1.41	1.91
**60**	63.03	5.05	61.10	1.83	9.95
**100**	105.07	5.07	99.42	0.58	24.85

When adding Rican noise to the signal prior to T2 fitting, the offset (method 4) increased for all T2, as both imperfect RF pulses and noise floor contribute to the offset in this case (Table 3). However, the performance of the four fitting methods (measured by δT2) was in the same order as for the noise-free simulations with method 4 performing most accurately and being superior to the other methods1 (Table 3). While the statistical error of all fitting methods is in the same range for low T2, methods 2 and 4 are more prone to statistical errors at high T2. This can be explained by the fact, that for long T2 the noise floor is not reached within the echo train, which increases the uncertainty in estimating offset and T2.

**Table 3:** Mean results of simulated signal with added noise fittings for each method per T2 time for an ESP of 5ms (ETL = 32, FA = 140°). Simulations were ran 1000 times.

**Method 1**			**Method 2**		
**T2(ms)**	**T2**_**fitted**_ **(ms)**	**dT2 (%)**	**T2**_**fitted**_ **(ms)**	**dT2 (%)**	**Offset**
**20**	22.06 ± 0.37	10.29 ± 1.82	21.70 ± 0.41	8.49 ± 2.05	3.40
**60**	64.51 ± 0.63	7.51 ± 1.05	66.31 ± 1.93	10.51 ± 3.21	-9.61
**100**	107.32 ± 1.01	7.32 ± 1.01	113.19 ± 5.58	13.1 ± 5.559	-26.11
**Method 3**			**Method 4**		
**T2(ms)**	**T2**_**fitted**_ **(ms)**	**dT2 (%)**	**T2**_**fitteds**_ **(ms)**	**dT2 (%)**	**Offset**
**20**	20.61 ± 0.45	3.05 ± 1.96	19.74 ± 0.5	1.30 ± 1.68	7.04
**60**	63.01 ± 0.66	5.01 ± 1.10	61.2 ± 1.943	2.05 ± 2.30	9.13
**100**	105.10 ± 1.05	5.10 ± 1.04	99.6 ± 4.884	0.36 ± 2.92	23.97

### Comparison of different fitting methods: Phantom measurements

**Fig 6:**
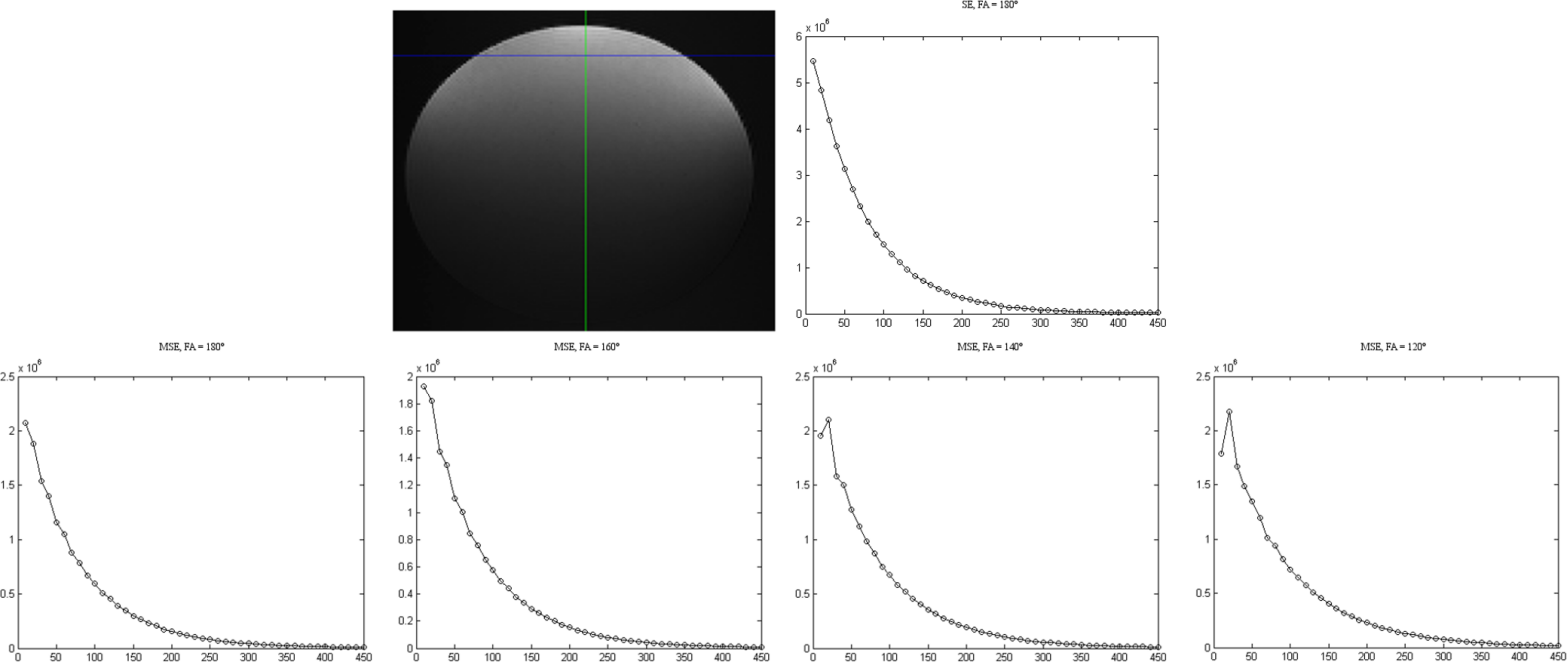
Example of the signal decay curves of the phantom measurements for a defined pixel (shown by the cross section of the lines): Curves are shown for the single spin echo sequence (top right) and the MSE sequences for different FAs (bottom row). The X-axis represents TE and y-axis the signal (x 10^6^ a.u). It can be noticed that as the FA reduces the first point, in particular, deviates from the expected exponential decay curve. The variation of the refocusing FA was performed by variation of the FA in the sequence protocol. The actual FA at the respective position might even differ from this value due to B1 inhomogeneities and imperfect slice profiles.

Signal decay in the phantom experiments (Fig 6) corresponded closely to the findings of the simulated work (Fig 2). Fitting of the data from the spin echo sequences (see Supporting Information, S1 Dataset: SE mapping) yielded a mean T2 of 67.7 ± 0.60 ms over three regions of interest (ROI) and over the four methods. Table 4 presents the mean T2 values and the relative deviation from T2, of three ROIs, determined from the spin echo sequences for all four fitting methods. Furthermore, for methods (2) and (4) the offset parameter is given.

**Table 4:** Fitted results from phantom measurements a MSE sequence with different FAs as used in the sequence protocol (see Supporting Information, S1 Dataset: MSE datasets) and different fitting methods. Single spin echo sequence yielded a mean T2 of 67.7 ± 0.60ms. T2 and dT2 relate to the mean and standard deviation over 3 ROIs within the phantom for each measurement.

	**Method 1**		**Method 2**		
**FA**	**T2 (ms)**	**dT2 (%)**	**T2 (ms)**	**dT2 (%)**	**Offset (a.u)**
**180°**	70.7 ± 1.0	3.5 ± 0.8	69.4 ± 1.2	1.9 ± 0.8	33949.6
**160°**	72.2 ± 0.9	5.6 ± 1.1	70.9 ± 1.1	4.1 ± 0.9	28239.8
**140°**	77.0 ± 0.7	12.7 ± 1.4	76.0 ± 0.9	11.6 ± 1.4	36127.6
**120°**	84.7 ± 0.6	24.0 ± 1.9	84.5 ± 0.8	24.1 ± 1.9	37770.0
	**Method 3**		**Method 4**		
**FA**	**T2 (ms)**	**dT2 (%)**	**T2 (ms)**	**dT2 (%)**	**Offset (a.u)**
**180°**	70.6 ± 1.0	4.6 ± 1.0	69.3 ± 1.1	3.6 ± 0.7	27230.2
**160°**	71.3 ± 0.9	5.5 ± 1.0	68.9 ± 1.2	2.8 ± 0.8	33127.8
**140°**	73.7 ± 0.8	9.1 ± 1.0	71.1 ± 1.1	6.2 ± 0.8	30207.4
**120°**	78.2 ± 0.8	15.7 ± 1.3	74.5 ± 1.1	11.3 ± 1.0	21556.9

Corresponding to our findings in the EPG simulations the relative deviation of T2 of the MSE from the reference value of the spin echo sequences increased with decreasing FA. Discarding the first echo reduced the relative T2 deviation for all FA. Relative T2 deviation was further reduced, if the offset was used as a fitting parameter (method 4). However, even for an 180° pulse as flip angle there still was a systematic error between 3.5% (method 1) and 3.6% (method 4) which is most likely due to an imperfect RF setup, B1 variations within the slice profile, or gross B1 inhomogeneity, which is known to occur particularly at high field strength [29].

## Discussion

Mono-exponential fitting is not the proper method for data fitting in T2 relaxometry due to its known inaccuracies in presence of B1 inhomogeneities. As mono-exponential fitting methods, however, are used in the majority of clinical and preclinical studies, our aim was to minimize the error in T2 by two modifications of mono-exponential fitting, that can be easily performed, are not time-intensive and have already been proposed: introducing an offset and/or discarding the first echo. By simulations based on EPG theory we could show, that B1 inhomogeneities and imperfect refocusing pulse angles provide major contributions to the *offset* fitting parameter in T2 relaxometry, while past published work pointed to the *offset* as being a compensation factor for either long T2 time within a compartment [13, 14, 19] or a baseline for noise and other system related signal [8, 20]. As the refocusing flip angle deviates from an optimal 180° pulse, the offset value increases. This can be explained by the fact, that the offset as a fitting parameter does only equal the asymptote of the signal curve, if the early echoes, i.e. the echoes at low TE, match the exponential decay curve exactly. If this is not the case, as for the example of oscillating between odd and even echoes, the vertical displacement from the perfect exponential decay curve contributes to the *offset*. The *offset* is particularly high for low ESP and low ETL, a setting which is often used particularly in T2 relaxometry in humans, as clinical MRI scanners usually limit the number of echoes and the user often reduces ESP to keep TR and thus, the acquisition time, as short as possible. and maintain the integrity of the curve for shorter T2 times (as measured in brain, muscle etc.).

Although we show that the *offset* is predominately due to an error in the refocusing FA, this does not mean, that noise and long T2 components do not play a role. Long T2 components, due to several proton pools with different T2 times or due to partial volume effects, always influence the determination of T2 and the *offset*, in addition to the effects of imperfect B1. If the longer T2 component is not covered by the entire ETL, then the *offset* would be higher. One could check for possible bi-exponential curves using a statistical Fχ test [30] and then fit the data as a bi-exponential function including the *offset* as an additional parameter [31].

Noise may also play a role in the final value of the offset, particularly in the lower values where there is a Rican and or Rayleigh distribution [32, 33]. It would be of interest to investigate the effect of SNR with the offset as a free parameter in the fitting.

Although the fitting offset parameter is strongly influenced by imperfections of the refocusing pulse angle, we found that using the *offset*, within method 2, as an additional fitting parameter does not lead to less systematic error in T2. Quite contrary, the systematic error in T2 increases by adding *offset* as fitting parameter, particularly for low ESP and low ETL, where the *offset* is high.

We have identified the signal oscillations in the early echoes as a major contribution to the *offset* and as a major source of error in T2quantification. One easy way to account for large portions of this error is to discard the first echo for curve fitting [34–36]. We, therefore, included this procedure as one possible post-processing method (method 3 in this publication) and found, that this method actually reduces the systematic error in T2, but most efficiently for high ESP. With low ESP, however, there is only a slight reduction in error, as the deviation of the first echo from the exponential signal decay curve is most prominent for high ESP as this allows for more mixing of longitudinal and transverse components at imperfect refocusing pulses.

It is important to note that the method of discarding the first echo will not be able to compensate for the systematic error in T2 completely, as all echoes are affected by imperfect refocusing pulses. Several authors have put forward that if there is a discrepancy in the refocusing FA, all odd echoes will not be completely refocused and all even echoes will refocus all isochromats into the transverse plane [37, 38]. From this they deduced the method to use only even echoes to determine the T2 time. Although this method allows one to get rid of large parts of the signal error, it does not eliminate the systematic error in T2 completely: As can be seen from the signal decay calculated with EPG theory in Fig 1, it is clear to see that although the curve with a true 180° pulse starts by following the even echoes, the curve matches closer to the odd echoes at the end. There is a mixing of the transverse and longitudinal components of the preceding echoes due to incorrect refocusing. That is, if the isochromats are not correctly refocused they will influence the subsequent echoes. This was further pointed out by Maudsley et. al. [39].

We could show, that most of the remaining systematic error in T2 after discarding the first echo can be eliminated by using the offset as a *fitting* parameter (method 4). This method works exceptionally well with low to intermediate ETL: δT2 was less than 6% for all ESP and all ETL≤32, even for a refocusing pulse of 120°. As in clinical as well as in experimental settings, most often relatively short echo train lengths are used, method 4 provides an easy-to-use, fast and reliable method to correct for B1 imperfections. Although it is thought that long ETL should be used to gain the most accurate determination of T2 [17] it is seen from Fig 3 that using long echo trains can be detrimental to the accuracy of T2, particularly when using method 4.

Although using the *offset* as a fitting parameter and discarding the first echo can minimize the error in T2, it should be pointed out that this method does not compensate errors in the refocusing pulse completely and further correction of the T2 may be of interest [12, 40]. In the first place, B1 inhomogeneities should always be reduced by advanced acquisition techniques, e.g. by using a larger spatial width of the refocussing pulse compared to the excitation pulse [14] or by parallel transmission [41]. A more recent example of a postprocessing algorithm to correct for the remaining B1 inhomogeneity would be the method introduced by Neumann et al. [11]. In this method the authors provide a means of correcting T2 with the use of a heuristic formula based on a lookup table derived from simulated EPG data. Although this method allows one to significantly reduce the systematic error in T2, there are several drawbacks: It needs much more computing time than the method introduced by us and is restricted to a limited choice of ETL (16, 24, and 32).

In conclusion, we have shown that the *offset* in T2 relaxometry is influenced by imperfect refocusing pulses. Using the *offset* as a fitting parameter and discarding the first echo is a fast and easy method to minimize the error in T2, particularly for low to intermediate echo train length. We expect that by identifying the optimal method for T2 determination, variations in reported times for specific tissue types and pathologies will be minimized, thus achieving better results for T2 as a quantitative measure.

## Supporting Information

S1 Dataset DICOM data sets from MRI investigations (Fig 6 and Table 4). (ZIP)
